# Influence of socioeconomic position and gender on current cigarette smoking among people living with HIV in sub-Saharan Africa: disentangling context from composition

**DOI:** 10.1186/s12889-016-3637-1

**Published:** 2016-09-20

**Authors:** Olalekan A. Uthman, Anna Mia Ekström, Tahereh T. Moradi

**Affiliations:** 1 Department of Public Health (IHCAR), Karolinska Institutet, Stockholm, Sweden; 2 Warwick-Centre for Applied Health Research and Delivery (WCAHRD), Division of Health Sciences, Warwick Medical School, The University of Warwick, Coventry, CV4 7AL UK; 3 Liverpool School of Tropical Medicine, International Health Group, Liverpool, Merseyside UK; 4 Department of Infectious Diseases, Karolinska University Hospital, Stockholm, Sweden; 5 Institute of Environmental Medicine, Division of Epidemiology, Karolinska Institutet, Stockholm, Sweden; 6 Centre for Epidemiology and Social Medicine, Health Care Services, Stockholm County Council, Stockholm, Sweden

## Abstract

**Background:**

Smoking is still gaining ground in Sub-Saharan Africa, especially among socially disadvantaged groups. People living with HIV represent a subgroup with a significantly elevated prevalence of cigarette smoking. The objective of the study was to examine the influence of individual-, neighbourhood- and country-level socioeconomic position on current cigarette smoking among people living with HIV in Sub-Saharan Africa.

**Methods:**

We applied multivariable multilevel logistic regression analysis on Demographic and Health Survey data collected between 2003 and 2012 in sub-Saharan Africa. We identified 31,270 individual living with HIV (Level 1) nested within 7,054 neighbourhoods (Level 2) from 19 countries (Level 3).

**Results:**

After adjustment for individual-, neighbourhood- and country-level factors, respondents, the following significant independent risk factors for increasing odds of being a current cigarette smokers among people living with HIV: male gender (odds ratio [OR] = 62.49; 95 % credible interval [CrI] 45.93 to 78.28), from the poorer households (OR = 1.62, 95 % CrI 1.38 to 1.90); living in urban areas (OR = 1.24, 95 % CrI 1.09 to 1.41), from neighbourhoods with low poverty rate (OR = 1.25, 95 % CrI 1.09 to 1.43), illiteracy rate (OR = 1.28, 95 % CrI 1.14 to 1.42), low unemployment rate (OR = 1.11, 95 % crI 1.01 to 1.43); and from countries with low socio-economic deprivation (OR = 1.53, 95 CrI 1.08 to 1.96). About 3.4 % and 39.4 % variation in cigarette smoking behaviour among people living with HIV is conditioned by differences between neighbourhoods and countries.

**Conclusions:**

Gender, education and socioeconomic context are independently associated with current cigarette smoking among people living with HIV in sub-Saharan Africa.

## Background

Tobacco use and HIV infection are two major causes of death globally that continue to grow [1]. The intersection of these two epidemics represents an area of growing clinical and public health importance, especially in sub-Saharan Africa. Living with HIV is associated with a two-fold increase in the likelihood of smoking cigarettes [2–4]. Individual living with HIV are more prone to the adverse health effects of tobacco use than those without [5–7]. In addition to the increased risk of numerous AIDS- and non-AIDS related disease, cigarette smoking has an adverse impact upon the health-related quality of life of people living with HIV [8]. Individual living with HIV in care loose more years of life to smoking than to HIV Infection [9].

Without objective and sound information about factors associated with cigarette smoking behaviours among people living with HIV, it will be difficult to plan effective care and strategies for smoking cessation. In the context of sub-Saharan Africa, much research has focused on individual compositional factors associated with cigarette smoking behaviours for both individuals living with HIV [10–13] and for general population [14–17]. It is well established that individual-level characteristics such as age, educational attainment, occupation and income are associated with smoking behaviours [10–17]. Indeed, we found no published studies that had examined contextual factors associated with current cigarette smoking among people living with HIV. This neglect is important given the central role of neighbourhoods in forming smoking habits [18–23], as they shape individual opportunities and expose residents to multiple risks and resources over the life course [24, 25]. The purpose of this study was to develop and test a model of factors associated with current cigarette smoking among people living with HIV that includes individual-level compositional socioeconomic characteristics along with contextual socioeconomic characteristics defined at the neighbourhood- and country level. We further, examined how much of the variation in cigarette smoking behaviour among people living with HIV is conditioned by differences between neighbourhoods and countries.

## Methods

### Study design and data

Data for this cross-sectional study were obtained from Demographic and Health Surveys (DHS), which are nationally representative household surveys conducted in low- and middle-income countries. This study used data from 19 recent DHS surveys conducted between 2003 and 2012 in sub-Saharan Africa available as of October 2014 and that included rapid HIV test results and questions on self-reported tobacco use. The DHS uses a multi-stage, stratified sampling design with households as the sampling unit [26]. Within each sample household, all women and men meeting the eligibility criteria are interviewed. Because the surveys are not self-weighting, weights are calculated to account for unequal selection probabilities as well as for non-response. With weights applied, survey findings represent the full target populations. The DHS surveys include a household questionnaire, a women’s questionnaire, and in most countries, a men’s questionnaire. All three DHS questionnaires are implemented across countries with similar interviewer training, supervision, and implementation protocols.

### HIV testing

For the HIV testing, blood spots were collected on filter paper from a finger prick and transported to a laboratory for testing. The laboratory protocol includes an initial enzyme-linked immunosorbent assay (ELISA) test, and then retesting of all positive tests and 5–10 % of the negative tests with a second ELISA. For those with discordant results on the two ELISA tests, a new ELISA or a Western Blot is performed [27]. Participation in the test was voluntary and before collecting blood samples each selected participant was asked to provide informed consent to the testing [27]. In order to ensure confidentiality, the HIV test results were anonymously linked to individual questionnaire information [27].

### Outcome variable

Respondents were explicitly asked “Do you currently smoke cigarettes?” Those who responded ‘yes’ to this question were defined as current cigarette smokers, whereas those who responded ‘no’ were defined as current non-smokers.

### Explanatory variables

#### Individual level factors

The following individual-level factors were included in the models: sex of the respondent (male versus female), respondents’ age in completed years (18 to 24, 25 to 34, 35 to 44 or 45 or older), educational attainment (no education, primary, secondary or higher); marital status (never married versus ever married) and occupation (working or not working). DHS did not collect direct information on household income and expenditure. We used DHS wealth index as a proxy indicator for socioeconomic position. The methods used in calculating DHS wealth index have been described elsewhere [28, 29]. Briefly, an index of economic status for each household was constructed using principal components analysis based on the following household variables: number of rooms per house, ownership of car, motorcycle, bicycle, fridge, television and telephone as well as any kind of heating device. From these criteria the DHS wealth index tertiles (poor, middle, and rich) were calculated and used in the subsequent modelling.

#### Neighbourhood-level factors

We used the term neighbourhood to describe clustering within the same geographical living environment. Neighbourhoods were based on sharing a common primary sample unit within the DHS data. The sampling frame for identifying primary sample unit in the DHS is usually the most recent census. The unit of analysis was chosen for two reasons. First, primary sample unit is the most consistent measure of neighbourhood across all the surveys [30], and thus the most appropriate identifier of neighbourhood for this cross-region comparison. Second, for most of the DHS conducted, the sample size per cluster meet the optimum size with a tolerable precision loss [31].

The following neighbourhood-level factors were included in the models: place of residence (rural or urban area), neighbourhood poverty-, illiteracy- and unemployment rates. We categorized neighbourhood poverty-, illiteracy- and unemployment rates into two categories (low and high), to allow for non-linear effects and provide results that were more readily interpretable in the policy arena. Median values served as the reference group for comparison.

### Country-level factors

Country-level data were collected from the reports published by the United Nations Development Program [32]. At country-level, we included percentage rural population and intensity of deprivation. Intensity of deprivation is average percentage of deprivation experienced by people in multidimensional poverty. Like wealth index, intensity of deprivation was computed using principal component based on data on household deprivations in education, health and living standards, however, at the country-level [32]. The country-level variables were also categorized into two (low and high) levels.

### Statistical analyses

#### Descriptive analyses

In the descriptive statistics the distribution of respondents by key variables were expressed as percentages.

#### Modelling approaches

**Fig. 1:**
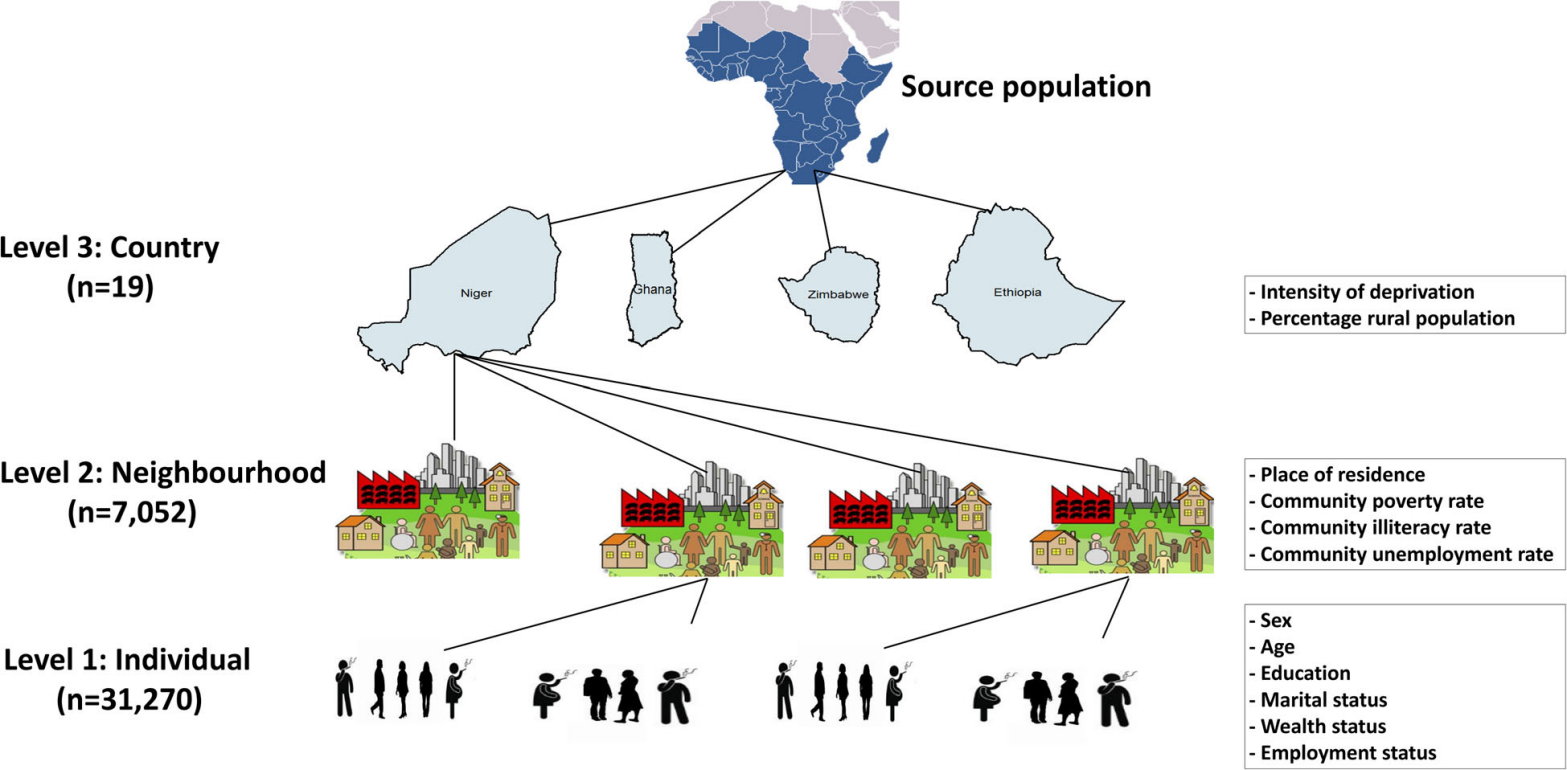
Multilevel data structure

We used multivariable logistic multilevel regression models to analyse the association between individual compositional and contextual factors associated with current cigarette smoking among people living with HIV. We specified a 3-level model for binary response reporting current cigarette smoking or not-currently smoking, for people living HIV (at level 1), in a neighbourhood (at level 2) living in a country (at level 3) (see Fig. 1).

We constructed five models. The first model, an empty or unconditional model without any explanatory variables, was specified to decompose the amount of variance that existed between country and neighbourhood levels. The second model contained only individual-level factors, the third model contained only neighbourhood-level factors, and fourth model contained only country-level factors. Finally, the fifth model simultaneously controlled for individual-, neighbourhood- and country-level factors (Full Model).

#### Fixed effects (measures of association)

The results of fixed effects (measures of association) were reported as odds ratios (ORs) with their 95 % credible intervals (CrIs). Bayesian statistical inference provides probability distributions for measures of association (ORs), which can be summarized with 95 % credible intervals (95 % CrI), rather than 95 % confidence intervals (95 % CI). A 95 % credible interval can be interpreted as there being a 95 % probability that the parameter takes a value in the specified range.

#### Random effects (measures of variation)

The possible contextual effects were measured by the intraclass correlation (ICC) and median odds ratio (MOR). We measured similarity between respondents in the same neighbourhood and within the same country using ICC. The ICC represents the percentage of the total variance in the probability of reporting current cigarette smoking that is related to the neighbourhood- and country-level, i.e. measure of clustering of odds of reporting cigarette smoking in the same neighbourhood and country. The ICC was calculated by the linear threshold (latent variable method) [33]. Following the ideas of Larsen et. al. on neighbourhood effects [34], we reported the random effects in terms of odds. The MOR measures the second or third level (neighbourhood or country) variance as odds ratio and estimates the probability of being a current cigarette smoker that can be attributed to neighbourhood and country context. MOR equal to one indicates no neighbourhood or country variance. Conversely, the higher the MOR, the more important are the contextual effects for understanding the probability of being a current cigarette smoker.

##### Model fit and specifications

We checked for multi-collinearity among explanatory variables examining the variance inflation factor (VIF) [35], all diagonal elements in the variance-covariance (*τ*) matrix for correlation between −1 and 1, and diagonal elements for any elements close to zero. None of the results of the tests provided reasons for concern. Thus, the models provide robust and valid results. The MLwinN software, version 2.31, was used for the analyses [36, 37]. Parameters were estimated using the Markov Chain Monte Carlo procedure [36]. The Bayesian Deviance Information Criterion was used as a measure of how well our different models fitted the data. A lower value on Deviance Information Criterion indicates a better fit of the model [38].

## Results

### Sample characteristics

The countries, year of data collection, and the surveys characteristics are listed in Table 1. The surveys were conducted between 2003 and 2012. The median number of neighbourhoods sampled was 569, ranging from 76 in Sao Tome and Principle to 9008 in Cote D’Ivoire. The median number of women and men living with HIV in the analysis was 370 and 199, respectively. The median prevalence of current cigarette smoking among women living with HIV was 0.6 %. The prevalence of current cigarette smoking among men living with HIV ranged from 10.3 % in Sao Tome and Principle to as much as 41.6 % in Lesotho. Table 2 presents the descriptive statistics for the final pooled sample. For this analysis, we analysed information on 31,270 people living with HIV (Level 1) nested within 7,052 neighbourhoods (Level 2) from 19 countries (Level 3) in sub-Saharan Africa. More than half of the respondents were female (58 %) and the majority of women were between the 18 and 34 years of age (60 %). Thirty-six percent of the respondents had no formal education. Most of the respondents were currently employed (70 %) and never married (81 %). Most of the respondents were living in rural.(60 %), high poverty rate. (68 %), high illiteracy rate. (61 %), and high unemployment rate. (55 %) neighbourhoods.

**Table 1:** Description of Demographic and Health Surveys data by countries, sex, smoking status and HIV prevalence in sub-Saharan Africa, 2003–2012

			Female				Male			
Country	Survey year	Number of neighbourhoods	Response rate	Sample size	HIV prevalence (%)	Cigarette smoker (%)	Response rate	Sample size	HIV prevalence (%)	Cigarette smoker (%)
Burkina Faso	2010	169	98.4	104	1.2	0.0	99.2	65	0.8	26.2
Burundi	2010	165	96.4	109	1.7	0.0	99.1	56	1.0	12.5
Cameroon	2011	653	97.3	435	5.6	1.1	98.9	218	2.9	24.8
Cote d’Ivoire	2011–12	9008	92.7	4656	4.6	0.2	97.7	4352	2.7	22.8
Ethiopia	2011	569	95.0	370	1.9	0.3	98.1	199	1.0	17.6
Gabon	2012	487	98.2	316	5.8	3.5	99.3	171	2.2	30.4
Ghana	2003	1358	95.7	540	2.7	0.2	98.7	818	1.6	13.1
Kenya	2008–09	472	96.3	318	8.0	0.9	97.7	154	4.6	24.7
Lesotho	2009	1540	97.9	997	26.7	0.6	97.6	543	18.4	41.6
Liberia	2013	210	97.6	147	2.0	2.7	99.4	63	1.7	12.7
Malawi	2010	1425	96.9	892	12.9	0.9	98.0	533	8.4	21.2
Niger	2012	8628	95.4	5102	0.4	0.0	98.1	3526	0.4	14
Rwanda	2010	420	99.1	266	3.7	1.5	99.7	154	2.2	23.4
Sao Tome and Principe	2008–09	76	89.8	37	1.3	2.7	94.2	39	1.8	10.3
Senegal	2010–11	93	92.7	61	0.8	0.0	98.2	32	0.5	15.6
Sierra Leone	2013	96	97.2	64	1.7	3.1	99.3	32	1.3	34.4
Swaziland	2006–07	2142	94.1	1438	31.1	2.1	94.8	704	19.7	24.7
Zambia	2007	1598	96.5	949	16.1	0.6	97.8	649	12.3	31.6
Zimbabwe	2010–11	5028	93.3	2782	17.7	0.3	96.0	2246	12.3	23.1

**Table 2:** Summary of pooled sample characteristics of the Demographic and Health Surveys data in sub-Saharan Africa, 2003–2012

Variable	Number (%)
Individual-level factors	31,270 (100)
Sex	
Female	18,016 (57.6)
Male	13,254 (42.4)
Age (in years)	
18–24	7,148 (22.9)
25–34	11,551 (36.9)
35–44	8,329 (26.6)
45+	4,242 (13.6)
Education	
No education	11,340 (36.3)
Primary	9,041 (28.9)
Secondary or higher	10,874 (34.8)
Wealth index	
Poorer	9,917 (31.7)
Middle	10,223 (32.7)
Richer	11,130 (35.6)
Employment status	
Not working	9.262 (29.6)
Current employed	22,008 (70.4)
Marital status	
Never married	6,100 (19.5)
Ever married	25,170 (80.5)
Neighbourhood-level factors	
Place of residence	
Urban	12,507 (40.0)
Rural	18,763 (60.0)
Poverty rate	
High	21,071 (67.4)
Low	10,199 (32.6)
Illiteracy rate	
High	19,130 (61.2)
Low	12,140 (38.8)
Unemployment rate	
High	17,141 (54.9)
Low	14,089 (45.1)
Country-level factors	
Percentage rural population	
High	18,952 (60.6)
Low	12,318 (39.4)
Intensity of deprivation	
High	9,567 (30.6)
Low	21,703 (69.4)

### Measures of associations (fixed effects)

The results of different models are shown in Table 3. In the fully adjusted model controlling for the effects of individual-, neighbourhood- and societal-level factors, men living with HIV were more likely to be current cigarette smokers than women living with HIV (OR = 62.49, 95 % CrI 45.93 to 78.28). Respondents with primary education were significantly more likely to be current cigarette smokers than those with secondary or higher education (OR = 1.38; 95 % CrI 1.24 to 1.53). Respondents from the poorer households were 62 % more likely to be current cigarette smokers as those from the richer households (OR = 1.62, 95 % CrI 1.38 to 1.90). Respondents currently working were significantly more likely to be current smokers than those not working (OR = 1.27, 95 % CrI 1.11 to 1.46). Respondents from urban areas were more likely to be current smokers than those from rural areas (OR 1.24; 95 % CrI 1.09 to 1.43). Respondents from low poverty (OR = 1.25, 95 % CrI 1.09 to 1.43) and illiteracy (OR = 1.28, 95 % CrI 1.14 to 1.42) rates neighbourhoods were more likely to be current cigarette smokers than those from high rates neighbourhoods. Finally, respondents from low intensity of deprivation were significantly more likely to be current cigarette smokers than those from high intensity of deprivation (OR = 1.53, 95 CrI 1.08 to 1.96).

**Table 3:** Individual compositional and contextual factors associated with cigarette smoking status among people living with HIV identified by multivariable multilevel logistic regression models, Demographic and Health Surveys data, 2003–2012

	Model 1^a^OR (95 % CrI)	Model 2^b^OR (95 % CrI)	Model 3^c^OR (95 % CrI)	Model 4^d^OR (95 % CrI)	Model 5^e^OR (95 % CrI)
Fixed-effect					
Individual-level factors					
Male (vs female)		59.06 (48.27, 75.98)			62.49 (45.93, 78.28)
Age (completed years)					
18–24		1 (reference)			1 (reference)
25–34		1.94 (1.69, 2.23)			1.95 (1.65, 2.22)
35–44		1.64 (1.39, 1.92)			1.64 (1.37, 1.90)
45+		1.29 (1.08, 1.53)			1.30 (1.06, 1.51)
Education					
No education		0.98 ((0.86, 1.11)			1.09 (0.95, 1.24)
Primary		1.34 (1.21, 1.50)			1.38 (1.24, 1.53)
Secondary or higher		1 (reference)			1 (reference)
Wealth index					
Poorer		1.21 (1.09, 1.35)			1.62 (1.38, 1.90)
Middle		1.09 (0.97, 1.20)			1.29 (1.12, 1.46)
Richer		1 (reference)			1 (reference)
Current (vs not) working		1.31 (1.14, 1.49)			1.27 (1.11, 1.46)
Never (vs currently) married		1.00 (0.88, 1.12)			1.02 (0.90, 1.14)
Neighbourhood-level factors					
Urban (vs rural)			1.21 (1.04, 1.42)		1.24 (1.09, 1.41)
Low (vs high) poverty rate			0.64 (0.52, 0.76)		1.25 (1.09, 1.43)
Low (vs high) illiteracy rate			0.82 (0.71, 0.95)		1.28 (1.14, 1.42)
Low (vs high) unemployment rate			0.84 (0.75, 0.95)		1.11 (1.01, 1.43)
Country-level factors					
Low (vs high) rural population				1.17 (0.84, 1.67)	1.23 (0.83, 1.77)
Low (vs high) intensity of deprivation				1.94 (1.44, 2.98)	1.53 (1.08, 1.96)
Random effects					
Country-level					
Variance (95 CrI)	0.18 (0.07, 0.40)	0.19 (0.08, 0.42)	0.14 (0.05, 0.31)	0.10 (0.03, 0.26)	0.20 (0.08, 0.45)
VPC (%)	3.4 (1.45, 7.0)	5.5 (2.4, 11.2)	2.6 (1.1, 5.2)	1.9 (0.6, 4.5)	5.8 (2.3, 11.8)
MOR (%, 95 % CrI)	1.50 (1.29, 1.85)	1.52 (1.31, 1.85)	1.43 (1.25, 5.17)	1.36 (1.18, 1.63)	1.54 (1.31, 1.90)
Explained variation (%)		−61.7	23.5	44.1	54.7
Neighbourhood-level					
Variance (95 CrI)	1.96 (1.70, 2.23)	0.01 (0.00, 0.02)	2.06 (1.81, 2.33)	1.99 (1.71, 2.28)	0.02 (0.01, 0.07)
VPC (%, 95 % CrI)	39.4 (35.1, 44.5)	5.8 (2.5, 11.6)	10.1 (36.2, 44.5)	38.9 (34.6, 43.6)	6.4 (2.5, 13.5)
MOR (%, 95 % CrI)	3.80 (3.47, 4.15)	1.10 (1.06, 1.13)	3.93 (3.62, 4.29)	3.84 (3.48, 4.22)	1.14 (1.08, 1.27)
Explained variation (%)		85.3	74.4	1.3	83.8
Model fit statistics					
DIC	18,237	14,988	18,160	18,231	14,931
Sample size					
Country-level	19	19	19	19	19
Neighbourhood-level	7,054	7,052	7,054	7,054	7,052
Individual-level	31,270	31,255	31,270	31,270	31,255

*OR* odds ratio, *CrI* credible interval, *MOR* median odds ratio, *VPC* variance partition coefficient, *DIC* Bayesian Deviance Information Criteria

^a^Model 1 – empty null model, baseline model without any explanatory variables (unconditional model)

^b^Model 2 – adjusted for only individual-level factors

^c^Model 3 – adjusted for only neighbourhood-level factors

^d^Model 4 – adjusted for only country-level factors

^e^Model 5 – adjusted for individual-, neighbourhood-, and country-level factors (full model)

### Measures of variations (random effects)

As shown in Table 3, in Model 1 (unconditional model), there was a significant variation odds of reporting current cigarette smoking across the countries (σ^2^ = 0.18, 95 % CrI 0.07 to 0.40) and across the neighbourhoods (σ^2^ = 1.96, 95 % CrI 1.70 to 2.23). According to the intra-country and intra-neighbourhood correlation coefficient, 3.4 % and 39.4 % of the variance in odds of reporting cigarette smoking could be attributed to the country- and neighbourhood-level factors, respectively. Results from the median odds ratio (MOR) also confirmed evidence of neighbourhood and societal contextual phenomena shaping individual cigarette smoking behaviour. From the full model (Model 5), it was estimated that if a respondent moved to another neighbourhood or another country with a higher probability of cigarette smoking, the median increase in their odds of being current cigarette smoker would be 1.14 (95 % CrI 1.08 to 1.27) and 1.54-fold (95 % CrI 1.31 to 1.90).

## Discussion

To our knowledge, the current study is the first multilevel examination of smoking behaviour among people living with HIV in sub-Saharan Africa using national representative data and a very large number of respondents (31,270). We found that at individual-level, respondents with primary education (vs those with secondary or higher), those currently working, and poorer households were significantly more likely to be current cigarette smokers. Gender appeared to be a very important risk factor, men living with HIV were significantly more likely to be current cigarette smokers than women living with HIV. The findings corroborate those of previous studies that examined the association between cigarette smoking and individual socioeconomic position both for people living with HIV [10–13] and the general population [14–17] in sub-Saharan Africa. More importantly, the findings uncover new evidence by demonstrating that neighbourhood- and country-level factors influence cigarette smoking behaviours above and beyond individual level factors.

Furthermore, our findings reveal a striking example of Simpson’s paradox [39, 40], a situation where the association between two variables (e.g. cigarette smoking and area poverty rate) are reversed when a third variable (e.g. individual wealth index) is considered. The results of including only neighbourhood-level factors showed that living in neighbourhood with low rates of socioeconomic position decreases the odds of being a current cigarette smoker. However, in the full model, when individual-, neighbourhood- and country-level factors were adjusted for simultaneously, there is a evidence that living in neighbourhood with low rates of socioeconomic position increases the odds of being a current cigarette smoker. The findings corroborate those of previous studies from high-income countries that had examined the effect of contextual effects on smoking in the general population [18–23].

We found evidence of geographical clustering in current cigarette smoking behaviours. About 3.4 % and 39.4 % of the variation in cigarette smoking behaviour among people living with HIV, is conditioned by differences between neighbourhoods and countries, respectively. If a respondent moved to another neighbourhood or another country with a higher probability of cigarette smoking, their odds of becoming a cigarette smoker may increase by about 14 % and 54 %, respectively. It is instinctual that people living with HIV from the same neighbourhood may be more similar to each other in relation to their current cigarette smoking behaviours than to others from other neighbourhoods [41], i.e. contextual phenomenon expresses itself as clustering of individual current cigarette smoking behaviours within neighbourhood. On these grounds, we might conclude that there is some evidence for a possible neighbourhood and country contextual phenomenon shaping a common individual cigarette smoking behaviours. These findings underscore the need to implement public health prevention strategies not only at the high-risk individual level, but also high-risk neighbourhoods, such as urban slum areas. In addition, there is a need for longitudinal studies to explain how deleterious behaviours are transmitted among individual residents, i.e. mechanisms that connect the people and the observed contextual factors. Similarly, further decomposition analyses could provide further evidence about important factors that could explain the disparities in cigarette smoking behaviour among high-risk individuals and high-risk places.

Our findings should be considered in light of the following limitations. Firstly, the cross-sectional nature of the data limits our ability to draw any causal inferences on the reported association reported. Secondly, we did not measure the length of time that participants had spent in their neighbourhoods and the extent of their exposure to the neighbourhood environment. We were, thus, unable to determine whether the associations of neighbourhood characteristics with current cigarette smoking were due to cumulated effects. Finally, one important limitation is that DHS surveys do not collect data on household income or expenditure, the traditional indicators used to measure wealth. The assets-based wealth index used here is only a proxy indicator of household economic status, and it does not always produce results similar to those obtained from direct measurements of income and expenditure where such data are available or can be collected reliably [28, 42]. In addition, we recognize potential data limitations that should be borne in mind while interpreting our findings. The observed magnitude of the association could have been under-estimated, since some African countries with huge populations and high HIV burden such as Nigeria could not be included in the model because of non-availability of comparable data. These countries were missing at not random, it is not clear why the DHS program did not include testing for HIV Infection in some countries.

Despite these limitations, the study strengths are significant. It is a large, population-based study with national coverage from 19 countries with high response rates. The DHS has some important advantages when compared with other surveys. They are often nationally representative, allowing for conclusions that cover the entire nation. In addition, variables in DHS were operationalized in the same way and making it possible for numerical values comparable across countries. There are advantages to studying factors associated with current cigarette smoking using a multilevel approach; we are able to provide more robust evidence about individual compositional and contextual measures of socioeconomic position associated with current cigarette smoking. The Bayesian approach we adopted has the additional advantage of being able to produce a far more robust estimate with better properties and yields unbiased estimates [43, 44]. Bryan and Jenkins state in their excellent discussion of this issue recommended [45]: “a third strategy would be to move beyond the classical (‘frequentist’) statistical framework used by most applied social science researchers and to make greater use of Bayesian methods of estimation and inference, as there is some evidence that they perform better in the mall number of countries.”

## Conclusions

In conclusion, individual compositional and contextual measures of socioeconomic position were independently associated with current cigarette smoking among people living with HIV in sub-Saharan Africa, which underscores the need to implement cigarette smoking prevention strategies not only at the individual level taking into account socioeconomic position, but also at the contextual levels.
